# Post-publication critique at top-ranked journals across scientific disciplines: a cross-sectional assessment of policies and practice

**DOI:** 10.1098/rsos.220139

**Published:** 2022-08-24

**Authors:** Tom E. Hardwicke, Robert T. Thibault, Jessica E. Kosie, Loukia Tzavella, Theiss Bendixen, Sarah A. Handcock, Vivian E. Köneke, John P. A. Ioannidis

**Affiliations:** ^1^ Department of Psychology, University of Amsterdam, Nieuwe Achtergracht 129-B, 1018 WT Amsterdam, The Netherlands; ^2^ Meta-Research Innovation Center at Stanford (METRICS), Stanford University, Stanford, CA, USA; ^3^ Departments of Medicine, Epidemiology and Population Health, Biomedical Data Science, and Statistics, Stanford University, Stanford, CA, USA; ^4^ School of Psychological Science, University of Bristol, Bristol, UK; ^5^ Department of Psychology, Princeton University, Princeton, NJ, USA; ^6^ School of Psychology, Cardiff University, Cardiff, UK; ^7^ Department of the Study of Religion, Aarhus University, Aarhus, UK; ^8^ Florey Department of Neuroscience and Mental Health, University of Melbourne, Melbourne, Australia; ^9^ Charité – Universitätsmedizin Berlin, Berlin, Germany; ^10^ Meta-Research Innovation Center Berlin (METRIC-B), QUEST Center for Transforming Biomedical Research, Berlin Institute of Health, Charité – Universitätsmedizin Berlin, Berlin, Germany

**Keywords:** peer review, post-publication critique, letter to the editor, meta-research, journal policy, scientific criticism

## Abstract

Journals exert considerable control over letters, commentaries and online comments that criticize prior research (post-publication critique). We assessed policies (Study One) and practice (Study Two) related to post-publication critique at 15 top-ranked journals in each of 22 scientific disciplines (*N* = 330 journals). Two-hundred and seven (63%) journals accepted post-publication critique and often imposed limits on length (median 1000, interquartile range (IQR) 500–1200 words) and time-to-submit (median 12, IQR 4–26 weeks). The most restrictive limits were 175 words and two weeks; some policies imposed no limits. Of 2066 randomly sampled research articles published in 2018 by journals accepting post-publication critique, 39 (1.9%, 95% confidence interval [1.4, 2.6]) were linked to at least one post-publication critique (there were 58 post-publication critiques in total). Of the 58 post-publication critiques, 44 received an author reply, of which 41 asserted that original conclusions were unchanged. Clinical Medicine had the most active culture of post-publication critique: all journals accepted post-publication critique and published the most post-publication critique overall, but also imposed the strictest limits on length (median 400, IQR 400–550 words) and time-to-submit (median 4, IQR 4–6 weeks). Our findings suggest that top-ranked academic journals often pose serious barriers to the cultivation, documentation and dissemination of post-publication critique.

## 1. Introduction

Poor quality research frequently survives peer review and permeates through to the academic literature [1–5]. This highlights the importance of ongoing critical scrutiny of published research to identify errors, limitations or alternative interpretations that were not adequately addressed during pre-publication peer review. Such critiques may help research consumers to make more informed judgements about the utility and validity of published scientific claims [6,7].

Journals exert considerable control over criticism of prior research submitted in the form of letters, commentaries or online comments [8,9]. We refer to these collectively as ‘post-publication critique' (see electronic supplementary material, SK for an operational definition). Currently, there is limited empirical data available to systematically evaluate how journals handle this important aspect of scientific self-correction. Prior studies of post-publication critique were narrow in scope, mainly focused on medical journals, and are now outdated [8,10–12]. In the present research, we sought to provide a systematic, cross-disciplinary and more contemporary assessment of journal policies (Study One) and practice (Study Two) related to post-publication critique at 330 top-ranked journals operating in 22 scientific disciplines (electronic supplementary material, SM provides a schematic illustration of the two studies). Our goal was to generate empirical evidence to inform debates about how scientific critique should be optimally handled by academic journals.

## 2. Study One

### 2.1. Methods

The study protocol (rationale, methods and analysis plan) was pre-registered on 14th February 2020 (https://osf.io/hjvnw/). All departures from this protocol are explicitly acknowledged in electronic supplementary material, SA. All data exclusions and measures conducted during this study are reported in this manuscript.

#### 2.1.1. Sample

The sample consisted of 15 academic journals, top-ranked by 2017 Journal Impact Factors operating in each of 22 scientific disciplines (*N* = 330 journals). This represents the entire population of interest. Journal Impact Factors were identified using Clarivate Journal Citation Reports (https://jcr.clarivate.com). Scientific disciplines were defined by Clarivate Essential Science Indicators: https://perma.cc/MD4V-A5X5; electronic supplementary material, SL). We did not include journals that only published reviews. The sample size was chosen based on a precision analysis which is documented in our preregistered protocol (https://osf.io/hjvnw/).

#### 2.1.2. Design

The study had a cross-sectional design. The measured variables (see electronic supplementary material, SB for details) were the name and description of any options for post-publication critique offered by each journal (e.g. letters), limits imposed on post-publication critique in terms of length (e.g. number of words), time-to-submit (e.g. weeks since publication of the target article) or number of references, and whether post-publication critiques are sent for independent external peer review (reviews solicited from individuals who were not members of the editorial team or authors of the target article). We also obtained 2017 Journal Impact Factors and identified whether journals were members of the Committee on Publication Ethics (COPE).

#### 2.1.3. Procedure

(1) Between November 2019 and January 2020, T.E.H. and V.E.K. identified and preserved the ‘article types' section of the author submission guidelines on each journal's website (electronic supplementary material, SC).
(2) Between February and August 2020, data extraction for each journal was performed independently by two authors using a Google Form (https://osf.io/bkvnw/) and instruction sheet (https://osf.io/5fmhb/). Authors were randomly assigned to 110 journals each as either first coders (S.A.H., T.B. and L.T.) or second coders (R.T.T., J.E.K. and T.E.H.) using the ‘sample' function in R.
(3) Coding was predominantly based on the preserved ‘article types' documentation to ensure stability and reproducibility (live journal websites can be updated). It was only necessary to examine live journal websites to check for web-based commenting systems. When an ‘article types' section was not found in step 1, each coder conducted an additional check of the live website and examined the most recently published issue of the journal to see if they could identify any examples of post-publication critique.
(4) Any coding differences were resolved through discussion between the assigned coders, with arbitration by an additional coder if necessary. If coding differences highlighted ambiguities in the extraction instructions, we discussed as a team, amended the instructions and adjusted any relevant prior coding to ensure alignment.
(5) If an article type seemed like it might be post-publication critique, but the description was insufficient to judge, coders checked several published articles of this type to determine whether any met our operational definition of post-publication critique (electronic supplementary material, SK).
(6) Because journals used various naming conventions for similar types of post-publication critique and various units to specify limits (e.g. characters, words or pages for length limits), we harmonized names to four types (letters [to the editor], commentaries, web comments and other) and transformed units to words (length limits) and weeks (time-to-submit limits). Letters and commentaries were types of articles submitted through the regular manuscript submission system, whereas web comments were submitted via an online interface on the target article webpage. For details, see electronic supplementary material, SD.

### 2.2. Results

#### 2.2.1. Journal characteristics

Across all 22 scientific disciplines (*n* = 330 journals), the median 2017 Journal Impact Factor was 7.39 (range 2.45–79.26). Two-hundred and thirty seven (72%) journals were members of COPE. Characteristics for each scientific discipline are available in electronic supplementary material, table SE1.

#### 2.2.2. How many journals offer post-publication critique?

**Figure 1.**
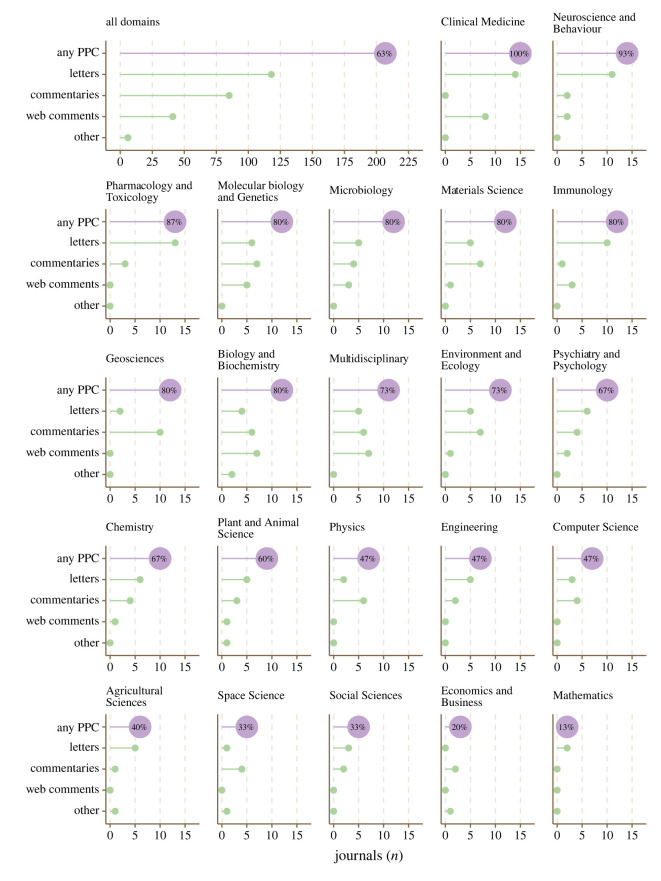
Number and percentage of journals offering any (purple) and specific (green) options for post-publication critique (PPC) in all scientific disciplines (top-left panel, *n* = 330 journals) and in individual scientific disciplines (other panels, *n* = 15 journals). Note that individual journals can offer multiple post-publication critique options.

Figure 1 shows the number of journals offering any form of post-publication critique, and specific types of post-publication critique, across 22 scientific disciplines (for equivalent tabular data, see electronic supplementary material, table SF1). Of 207 journals that offered post-publication critique, 154 (74%) were members of COPE. Of 123 journals that did not offer post-publication critique, 83 (67%) were members of COPE.

Journals offering post-publication critique were most common in Clinical Medicine (*n* = 15, 100%) and least common in Mathematics (*n* = 2, 13%). Overall, 166 journals offered one option for post-publication critique, 39 journals offered two options and two journals offered three options, equating to a total of 250 individual post-publication critique options across journals.

After post-publication critique names were harmonized into four types, there were 118 journals offering letters, 85 journals offering commentaries and 41 journals offering web comments. Six journals offered other miscellaneous types of post-publication critique such as ‘Forum papers' and ‘Update articles'. A complete list of journals and their post-publication critique options is available in electronic supplementary material, table SG1.

#### 2.2.3. What limits did journals place on post-publication critique?

**Figure 2.**
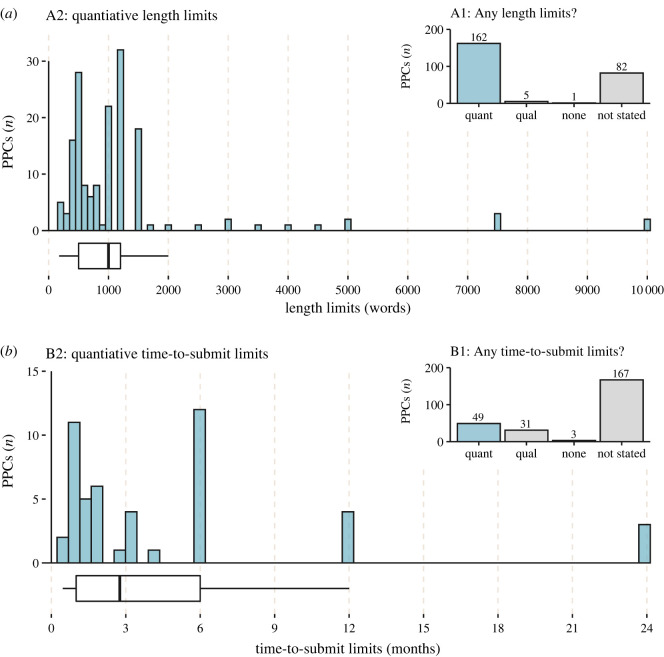
Limits imposed by journals on post-publication critique (PPC) in terms of (*a*) length and (*b*) time-to-submit since publication of the target article. A1 and B1 show the number of post-publication critique options for which the journal did not state if there was a limit (Not stated), explicitly stated there was not a limit (None), stated a qualitative limit (Qual) or stated a quantitative limit (Quant). Quantitative limits are displayed in A2 and B2 as a histogram and boxplot with the dark line representing the median, lower and upper hinges representing the 25th and 75th percentiles, and upper and lower whiskers representing the ±1.5 interquartile range.

Table 1 shows how often journal policies imposed limits on post-publication critique in terms of length, time-to-submit or number of references. Limits were mostly expressed quantitatively, but sometimes they were qualitative and more ambiguous, for example, stating that post-publication critique should be ‘concise' or address ‘recently published' articles. Often there was no information at all about a particular limit. Occasionally policies explicitly asserted that there was no limit. This happened once for length limits, three times for time-to-submit limits and 21 times for reference limits. The full distribution of quantitative length limits and time-to-submit limits is displayed in figure 2 and limits imposed by individual journals are available in electronic supplementary material, table G1. Table 1 also shows whether post-publication critique was subject to independent external peer review (for details, see electronic supplementary material, table SH1).

**Table 1.** Post-publication critique types and their length, time-to-submit, or reference limits. The table shows the number (*n*) and percentage (%) of post-publication critique types that are subject to any (qualitative or quantitative) limit, quantitative limits specifically, and the median (Md) and interquartile range (IQR) for quantitative limits. The table also shows whether the author submission guidelines state that the post-publication critique types are sent for independent external peer review either routinely or at the editor's discretion (for details see electronic supplementary material, SH).

post-publication critique type	length limits			time-to-submit limits			reference limits			peer review
	any	quantitative		any	quantitative		any	quantitative		
	*n (*%)	*n* (%)	Md words (IQR)	*n* (%)	*n* (%)	Md weeks (IQR)	*n* (%)	*n* (%)	Md refs (IQR)	*n* (%)
letters (*n* = 118)	92 (78%)	92 (78%)	500 (513)	50 (42%)	39 (33%)	8 (22)	68 (58%)	65 (55%)	6 (5)	24 (20%)
commentaries (*n* = 85)	62 (73%)	59 (69%)	1200 (300)	25 (29%)	10 (12%)	26 (26)	41 (48%)	38 (45%)	15 (0)	47 (55%)
web comments (*n* = 41)	11 (27%)	10 (24%)	600 (75)	5 (12%)	0 (0%)	—	2 (5%)	2 (5%)	8 (3)	0 (0%)
other (*n* = 6)	2 (33%)	1 (17%)	10 000 (0)	0 (0%)	0 (0%)	—	1 (17%)	0 (0%)	—	3 (50%)
all types (*n* = 250)	167 (67%)	162 (65%)	1000 (700)	80 (32%)	49 (20%)	12 (22)	112 (45%)	105 (42%)	10 (10)	74 (30%)

Because some journals offered more than one type of post-publication critique, figure 2 does not give a complete picture of journal-level limits. For example, an individual journal may compensate for a restrictive form of post-publication critique by also offering a less restrictive form. This is difficult to assess systematically across the whole sample because of interactions between different limit types and the ambiguity of qualitative limits. However, in table 2, we provided a focused examination of the 20 journals that offered the most restrictive post-publication critique options in terms of quantitative length and time-to-submit limits. To build this table, we created two separate lists of post-publication critique ranked by quantitative length limits and time limits, respectively. We then identified the top 10 journals in each ranked list. To handle duplicates within- or between- lists, we retained the higher ranked instance and replaced the lower-ranked instance until we had 20 unique journals overall (table 2).

**Table 2.** Twenty journals that offered the most restrictive options for post-publication critique. Journals were selected based on having the most restrictive quantitative length and time-to-submit limits for at least one of their post-publication critique options. Some of these journals also offered additional less restrictive options, which we have included and marked with asterisks. When post-publication critiques were subject to qualitative limits, the verbatim policy text is shown. Journals are presented in alphabetical order. Journal of the American Medical Association (*JAMA*) journals are clustered because they had identical post-publication critique policies.

journal	post-publication critique type	length limit (words)	time-to-submit limit
Annals of Internal Medicine	letters	400	four weeks
	web comments^a^	Not specified	not specified
Annals of Neurology	letters	400	not specified
Clinical Pharmacology and Therapeutics	letters	400	six months
Emerging Infectious Diseases	letters	300	four weeks
	web comments^a^	1667	not specified
JAMA, JAMA Internal Medicine, JAMA Neurology, JAMA Oncology, JAMA Psychiatry	letters	400	four weeks
	web comments	600	'We may reject comments because they…are submitted a long time after article publication'
Journal of the Royal Statistical Society Series B-Statistical Methodology	letters	400	1 year
Lancet	letters	250	two weeks
Lancet Diabetes and Endocrinology	letters	400	eight weeks
Lancet HIV	letters	250	four weeks
Lancet Infectious Diseases	letters	400	six weeks
Lancet Psychiatry	letters	400	four weeks
Lancet Respiratory Medicine	letters	400	four weeks
National Science Review	letters	300	not specified
Neurology	letters	200	'…restricted to comments about studies published in Neurology within the past eight weeks, with the exception of submissions identifying possible errors in data or data analysis, or by appeal to the Editor'
New England Journal of Medicine	letters	175	three weeks
	web comments	200	not specified
Science	letters	300	three months
	web comments^a^	‘…brief…’	not specified
	commentaries^a^	1000	three months

^a^Journals are included in the table based on having the most restrictive post-publication critique options; however, for each journal, we have also included all other options for post-publication critique they offered and marked them with asterisks.

From table 2, it is notable that eight of the journals offering the most restrictive options for post-publication critique operate in the discipline of Clinical Medicine. Overall, medical journals specified strict limits on length (19 policies with a quantitative limit: median 400, IQR 150 words; three policies did not mention a limit) and time-to-submit (13 policies with a quantitative limit: median 4, IQR 2 weeks; three policies stated a qualitative limit, five policies did not mention a limit, one policy stated there was no limit). Table 2 also suggests that restrictive options for post-publication critique are sometimes accompanied by less restrictive options. Eleven of the 20 journals only offered one option for post-publication critique. Nine of the 20 journals offered web comments in addition to letters and, in general, web comment policies appeared to be less restrictive than letters. However, this was often unclear because exact quantitative limits were not specified and most differences were marginal. For example, in the *JAMA* family of journals, letters must be less than 400 words and submitted within four weeks of target article publication. By contrast, web comments are marginally less restrictive in terms of length (600 words), and ambiguous about their time-to-submit limits (We may reject comments because they … are submitted a long time after article publication). One journal—*Science*—offered commentaries (called ‘Technical Comments') in addition to letters and web comments. In this case, letters and commentaries shared a time-to-submit limit of three months, and commentaries had a somewhat less restrictive length limit than letters (1000 versus 300 words). By contrast, no time-to-submit limit was specified for web comments and an ambiguous length limit was implied (web comments should be ‘brief').

## 3. Study Two

### 3.1. Methods

The study protocol (rationale, methods and analysis plan) was pre-registered on 14th February 2020 (https://osf.io/hjvnw/). All departures from this protocol are explicitly acknowledged in electronic supplementary material, SA. All data exclusions and measures conducted during this study are reported in this manuscript.

#### 3.1.1. Sample

The sample consisted of 10 randomly sampled eligible articles published in 2018 for each of the 207 journals that offered post-publication critique in principle (according to the results of Study One), aside from one journal, *Wildlife Monographs*, which only published six articles in 2018. Thus, the sample size was 2066 articles.

To obtain this sample, one author (T.E.H.) downloaded bibliographic records from Clarivate Web of Science for all articles published in each journal offering post-publication critique in 2018. We did not include records with meta-data indicating that the article was a ‘Correction', ‘Retraction', ‘News Item', ‘Book Review', ‘Meeting Abstract' or ‘Biographical-Item’. The remaining records were randomly shuffled using the ‘sample' function in R. During manual inspection, additional articles were excluded if they (i) could not be found or accessed; (ii) were non-English language; (iii) had been retracted or (iv) did not include substantive research: specifically, we excluded news, book reviews, editorials, previews or similar, and included empirical research, case studies, simulations, proofs, theoretical papers, reviews, meta-analyses and perspectives (if they were predominantly evidence-based rather than opinion-based). If articles were themselves examples of post-publication critique, they were also excluded for the purposes of our primary prevalence measure, but included for the purposes of our secondary prevalence measure.

#### 3.1.2. Design

The study had a cross-sectional design. The goal of Study Two was to examine post-publication critique in practice at the 207 journals that offered post-publication critique according to Study One. We used two measures of post-publication critique prevalence. Our primary (preregistered) estimate of prevalence was based on how many of 10 randomly sampled articles per journal were linked to post-publication critique. As one journal—*Wildlife Monographs*—only published six articles in 2018, the total number of assessed articles was 2066. An article was considered linked to post-publication critique if the article webpage mentioned the existence of relevant post-publication critique.

After Study One, but before beginning Study Two, we decided to also compute a secondary (not preregistered) prevalence estimate based on how many of the randomly sampled articles were themselves examples of post-publication critique. To align the two estimates, we only used the first 10 eligible articles for each journal, and therefore the total number of assessed articles was 2066 as above. These two prevalence measures have complementary strengths and limitations. For the primary prevalence estimate, post-publication critique was identified through searches of article web pages for linked post-publication critique. Its accuracy therefore depends on journals actively and visibly linking to post-publication critique on their webpages. However, it is not dependent on post-publication critique being indexed in Web of Science databases. By contrast, for the secondary prevalence estimate, post-publication critique was identified by checking if sampled articles were themselves examples of post-publication critique. Thus, it does not rely on journals linking to relevant post-publication critique, but it does rely on post-publication critique being indexed in Web of Science databases, because that is how the sampled articles were identified. Note that our primary estimate was also time-restricted in the sense that any identified post-publication critique must have been published after 2018 (when the sampled articles were published). By contrast, our secondary estimate could theoretically detect post-publication critiques pertaining to any prior articles, regardless of their publication date.

We also examined several variables related to how post-publication critique was conducted in practice. This assessment was only performed on post-publication critiques we identified via the primary prevalence measure. For each post-publication critique, we categorized the type of issues that were addressed (design, implementation, analysis, reporting, interpretation or other), length (in words), whether new data were collected, whether novel analyses were performed, time since publication of the target article (in days), open access status of target article and post-publication critique, whether post-publication critique included a conflict of interest statement and whether it declared any conflicts of interest, whether post-publication critique authors were anonymous, whether the post-publication critique triggered a correction to the target article, whether target article authors replied, and if they replied, whether they collected new data or performed novel analyses, and whether they stated that their core claims remained unchanged after reading the post-publication critique. For more detail about variables measured in Study Two, see electronic supplementary material, table SI.

#### 3.1.3. Procedure

(1) Between May 2021 and September 2021, each coder (S.A.H., T.B., R.T.T., J.E.K., L.T. or T.E.H.) self-assigned journals sequentially from a randomly shuffled list until they had coded an approximately equal amount. For each journal, the assigned coder worked sequentially through a list of randomly shuffled articles published by that journal in 2018. If a coder did not have access to a journal, it was skipped and assigned to the next available coder.
(2) For each article, coders ascertained whether it (i) needed to be excluded; (ii) was itself an example of post-publication critique; or (iii) was linked to post-publication critique. When we encountered multiple post-publication critiques that were part of the same back-and-forth exchange between target article authors and post-publication critique authors, these were counted as a single instance of post-publication critique. Coders followed an instruction sheet (https://osf.io/aejx4/), which reminded them of the exclusion criteria and operational definition of post-publication critique (electronic supplementary material, SK), and entered data directly into a Google Sheet. Coders were encouraged to discuss any ambiguous cases with T.E.H. and all positive post-publication critique classifications were independently verified by T.E.H.
(3) When journals relied on third-party services (e.g. Elsevier's ScienceDirect) to distribute their articles, we only used these websites if the journal did not also distribute their articles through their own dedicated website (as we noted that links to post-publication critique were sometimes displayed on journal websites and not on third-party websites).
(4) Coders worked sequentially through each journal's articles until they had examined 10 that were eligible (i.e. they were not excluded or classified as themselves being post-publication critique).
(5) For the assessment of post-publication critique features, one author (T.E.H.) extracted information (see electronic supplementary material, table SI) from all linked post-publication critiques identified by the primary estimate method.

#### 3.1.4. Data analysis

For prevalence estimates, 95% Wilson Confidence intervals computed by the R function ‘prop.test' are reported in square brackets.

### 3.2. Results

#### 3.2.1. How prevalent is post-publication critique in practice?

In total, we considered 3030 articles for inclusion before we reached our target of 2066 eligible research articles. Seven-hundred and ninety-one articles were excluded because they did not contain research (*n* = 770) or could not be found/accessed (*n* = 21). An additional 173 articles were classified as being themselves examples of post-publication critique and excluded from our primary prevalence estimate. In total, 39 of the 2066 research articles were linked to at least one post-publication critique. Our primary post-publication critique prevalence estimate was therefore 1.9% [1.4, 2.6]. These articles were published in 22 individual journals (electronic supplementary material, table SJ2).

We also computed a secondary prevalence estimate based on the proportion of articles that were themselves post-publication critique among the first 10 eligible articles assessed at each journal (first six articles in the case of *Wildlife Monographs; N* = 2066 articles). We only examined the first 10 eligible articles in order to align the denominator of the primary and secondary estimates. This meant that articles classified as being themselves examples of post-publication critique only contributed if they were found within the first 10 eligible articles, and thus, we did not include all of the 173 post-publication critiques found among the 3030 articles mentioned above. One-hundred and fifteen out of the 2066 articles were classified as post-publication critique, yielding a secondary prevalence estimate of 5.6% [4.6,6.7]. This is equivalent to 5.9 post-publication critiques for every 100 research articles. These articles were published in 52 individual journals (electronic supplementary material, table SJ3).

**Figure 3.**
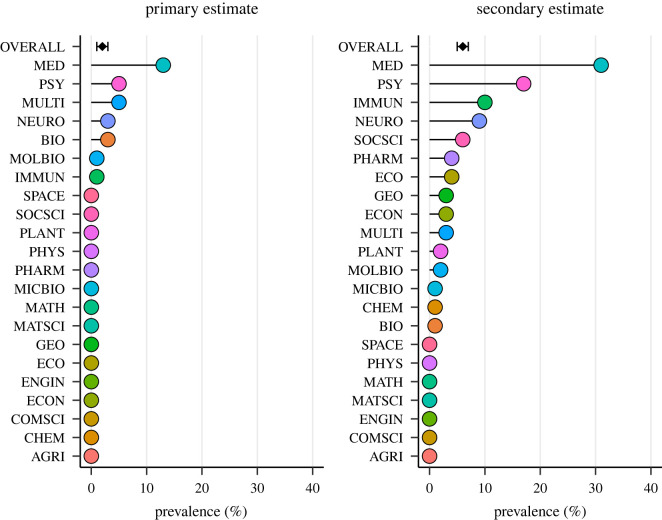
Primary (*a*) and secondary (*b*) prevalence estimates for post-publication critique in all journals overall (*N* = 330 journals; black diamond, error bars represent 95% confidence intervals) and then in descending order by each scientific discipline (*n* = 15 journals; coloured circles). Discipline abbreviations: Agricultural Sciences (AGRI), Biology and Biochemistry (BIO), Chemistry (CHEM), Clinical Medicine (MED), Computer Science (COMSCI), Economics and Business (ECON), Engineering (ENGIN), Environment and Ecology (ECO), Geosciences (GEO), Immunology (IMMUN), Materials Science (MATSCI), Mathematics (MATH), Microbiology (MICBIO), Molecular Biology and Genetics (MOLBIO), Multidisciplinary (MULTI), Neuroscience and Behaviour (NEURO), Pharmacology and Toxicology (PHARM), Physics (PHYS), Plant and Animal Science (PLANT), Psychiatry and Psychology (PSY), Social Sciences (SOCSCI), Space Science (SPACE).

Prevalence estimates varied substantially across scientific disciplines (figure 3; for equivalent tabular data see electronic supplementary material, table SJ1). The number of disciplines which had zero instances of post-publication critique was 15 according to our primary prevalence estimate and 7 according to our secondary prevalence estimate. The discipline with the most post-publication critique was Clinical Medicine with 19 (13%) of 150 research articles being linked to post-publication critique and 47 (31%) of 150 assessed articles being themselves instances of post-publication critique.

#### 3.2.2. Features of post-publication critique in practice

We conducted a closer examination of the post-publication critique linked to the 39 articles identified above for our primary prevalence estimate. These 39 articles were each linked to either one (*n* = 27), two (*n* = 7), three (*n* = 3) or four post-publication critiques (*n* = 2); a total of 58 post-publication critiques. Various features of these post-publication critiques are shown in table 3 and features of target article author responses to these post-publication critiques are shown in table 4.

**Table 3.** Features of 58 assessed post-publication critiques. NC, not calculable (because *n* = 1).

*post-publication critique type*	*n*			
commentaries	1			
web comments	13			
letters	44			
*journals*	*n*			
New England Journal of Medicine	18			
JAMA	6			
Lancet Psychiatry	6			
The BMJ	4			
Lancet Oncology	3			
Current Biology	2			
Gastroenterology	2			
Neurology	2			
Science	2			
13 other journals	1			
*disciplines*	*n*			
Clinical Medicine	35			
Psychiatry and Psychology	8			
Multidisciplinary	5			
Biology and Biochemistry	4			
Neuroscience and Behavior	4			
Immunology	1			
Molecular Biology and Genetics	1			
*open access to target article and post-publication critique*	*n*			
target paywalled, post-publication critique paywalled	22			
target public, post-publication critique public	19			
target paywalled, post-publication critique public	10			
target public, post-publication critique paywalled	7			
*post-publication critique author anonymity*	*n*			
not anonymous	57			
anonymous	1			
*post-publication critique conflict of interest* (*COI*)	*n*			
statement declares no COI	30			
statement declares COI	15			
no COI statement	13			
*type of issues raised in post-publication critique*	*n*			
design	19			
implementation	3			
analysis	19			
reporting	10			
interpretation	45			
ethics (other)	1			
*data availability in target article*	*n*			
no data availability statement	43			
data sharing not applicable	7			
statement says data are available upon request	5			
statement says data are available	3			
*analyses/data included in post-publication critique*	*n*			
no analyses or data	51			
novel analyses of new data	5			
novel analyses of original data	1			
novel analyses of original and new data	1			
*length of post-publication critique* (*words*)				
*post-publication critique type*	*md*	*min*	*max*	*IQR*
letters	251	104	1420	177
commentaries	1586	1586	1586	NC
web comments	108	9	993	309
all types	251	9	1586	199
*time of post-publication critique publication relative to target article publication* (*days*)				
*post-publication critique type*	*md*	*min*	*max*	*IQR*
letters	133	21	1053	86
commentaries	525	525	525	NC
web comments	63	5	1066	789
all types	133	5	1066	112

**Table 4.** Features of target article author response to 58 assessed post-publication critiques.

*Did the post-publication critique prompt publication of a correction?*	*n*
no	56
yes	2
*Did the post-publication critique prompt publication of an author reply?*	*n*
yes	44
no	14
*Did the authors' reply involve collection of new data?*	*n*
no	42
yes	2
*Did the authors' reply involve new analyses?*	*n*
no	40
yes	4
*Did the authors' reply assert that their core claims remain unchanged?*	*n*
yes	41
no	3

### 3.3. Discussion

We found substantial variation in how post-publication critique was handled in both policy and practice at 330 top-ranked journals operating in 22 scientific disciplines. Post-publication critique was rare in most disciplines and a considerable number of journals (37%) did not offer any options for submitting post-publication critique. Journals that did offer post-publication critique often imposed restrictive length and time-to-submit limits. Overall, top-ranked journals often represented a serious obstacle to the cultivation, documentation and dissemination of post-publication critique.

There was substantial variation across scientific disciplines with journals in Clinical Medicine standing out as offering the most options for post-publication critique and publishing the most post-publication critique, but also imposed the most restrictive length and time-to-submit limits (for concordant evidence, see [8,10–12]. In general, health-related disciplines seemed to have a more active post-publication critique culture than non-health-related disciplines like the physical sciences and social sciences. Many disciplines, such as mathematics, showed little evidence of any post-publication critique activity, with few journals offering post-publication critique and scarce evidence of published post-publication critique. Our data do not speak to the causal forces that underlie these inter-discipline differences, but some potential contributing factors could be cultural (e.g. different attitudes towards scientific criticism and how it should be handled), pragmatic (e.g. differences in methodological standards and research quality, manifesting in differential need for scientific criticism), bureaucratic (e.g. different resources available to support post-publication critique) or historic (e.g. individuals or events that highlighted the value of post-publication critique).

Post-publication critique could usually be mapped to one of three main types (letters, commentaries and web comments), of which letters were most common in policy and practice. Generally, letters had the most restrictive limits, followed by commentaries, then web comments. Typically, letters had to be shorter, submitted more quickly, and contain fewer references relative to commentaries. Usually, web comments had no stated limits, except for a quarter that had length limits. Policies implied that commentaries were more likely to be sent for independent external peer review, with letters more likely to be handled exclusively by the editorial team. Web comments were typically subject to ‘light' editorial moderation or no review at all. Some journals may offer less restrictive web comments to compensate for other more restrictive options for post-publication critique they offer (table 2).

The extent to which journal limits on post-publication critique are reasonable or unreasonable is a somewhat subjective determination and there are likely to be competing interests between what is best for journals and what is best for the advancement of science. Restrictions on post-publication critique may arise from editorial bias against criticism of papers they have published. Editors may also prefer to allow only what they perceive as the most timely and concise debate. However, length restrictions arbitrarily limit the scope of post-publication critique, particularly if the criticism involves extended analyses or additional data. One can certainly say very little of substance in 175, 200 or 250 words (the most restrictive length limits). Restricting the number of references to 3, 4 or 5 (the most restrictive reference limits) may prevent links to relevant evidential, contextual or methodological information, undermining an aspect of scholarship that is surely as important to post-publication critique as it is to regular articles. Finally, imposing time-to-submit limits on post-publication critique is clearly not justifiable from a scientific perspective because important critiques may arise at any time. Limiting the time allowed to submit post-publication critique to two, three or four weeks (the most restrictive time-to-submit limits) seems especially unjustifiable and poses a serious threat to the dissemination of scientific critique. An earlier study describing strict length and time-to-submit limits imposed on post-publication critique at six leading medical journals led the author to trenchantly conclude that ‘In effect, there is a statute of limitations by which authors of articles in these journals are immune to disclosure of methodological weaknesses once some arbitrary (short) period has elapsed, which cannot be right' ([8]; also see [6]).

Our exploration of how post-publication critique is used in practice suggested that letters are far more common than other types of post-publication critique, perhaps because they are the most frequently available post-publication critique option and also because, as formal articles, they may impart greater academic credit to their authors than informal web comments (table 3). We found that half of the post-publication critiques we examined were behind a paywall, sometimes even when the target article was publicly accessible. This reduces access to post-publication critique for both professional scientists and other readers, like patients, journalists and policy-makers [9]. Most post-publication critiques had conflict of interest statements and a third of those statements declared a potential conflict. Conflict of interest statements enable readers to evaluate an important risk of bias and seem just as relevant to post-publication critiques as they are to other academic articles [13].

The post-publication critiques we examined addressed a range of issues spanning the timeline of a research project, including design, implementation, analysis, reporting and interpretation (for concordant evidence, see [10]). The vast majority of post-publication critiques did not include new analysis of original or new data. This may be because very few original articles stated that data were available, as is typical in many scientific disciplines [14–16]. Most of the post-publication critiques were short (approx. 250 words) and published within five months of the target article, perhaps partly because they were published in some journals that imposed the strictest limits on post-publication critique (table 2).

In the majority of cases, target article authors replied to post-publication critique, particularly for letters or commentaries relative to web comments. Author replies rarely included new data or analyses. We found that only two post-publication critiques prompted publication of a correction and in all but three cases, the target article authors asserted that their core claims remained unchanged despite the arguments presented in the post-publication critique. It was beyond the scope of our study to examine whether author replies were appropriate and justified, but prior research has suggested that they are often inadequate [17]. In all, target article authors seemed almost entirely immune to the criticisms raised, with rare exceptions.

Our two studies have some important limitations. Firstly, we believe our operational definition of post-publication critique (electronic supplementary material, SK) captures the most explicit journal-based avenues for scientific criticism, but it will inevitably miss indirect or less formal critique as is, for example, embedded in research or review articles with a broader focus, or as occurs outside of journals (e.g. on social media or external commenting platforms, such as PubPeer). We also did not include errata, corrections, corrigendums, retractions or similar, in our definition, though such notifications can be prompted by peer scrutiny (e.g. [18,19]). Adopting a precise definition was necessary to ensure clarity and tractability. Secondly, for Study One we relied on information as stated on journal websites as of November 2019, and for Study Two, we relied on a random sample of articles published in 2018. Our assessment therefore cannot account for incomplete policy statements, more recent policy updates, or unpublished information, such as numbers of post-publication critiques rejected, modified, or delayed. Because of a lack of consistency and clarity in the presentation of post-publication critique policies, we were unable to reliably extract information on other potentially interesting features, such as fees to submit or publish, or whether post-publication critique is routinely indexed in academic databases. Thirdly, we focused on a sample of top-ranked journals only and it is unclear to what extent our findings may extend to other journals. For example, it may be that more recently established journals are more progressive and open to critical scrutiny of their publications compared to top-ranked journals. Articles published in lower-ranked journals may also receive less attention overall, and thus receive even less post-publication critique. Fourthly, there is no objective method for delineating scientific disciplines, which are often porous and overlapping. All categorization schemes therefore have limitations. We opted to use an established categorization schema provided by Essential Science Indicators, but there is an element of arbitrariness to the assignment of journals to disciplines. For example, JAMA Psychiatry is assigned to the discipline of Psychology & Psychiatry, but could arguably also be assigned to Clinical Medicine.

Many of our findings imply that the extant culture of journal-based post-publication critique is suboptimal, though more detailed scrutiny of policies and practice at specific journals will enhance this diagnosis. It is interesting to note that of the 123 journals that did not offer any options for submitting post-publication critique, 83 were members of COPE, an organization whose guidelines state that ‘Journals must allow debate post publication either on their site, through letters to the editor, or on an external moderated site, such as PubPeer' [20].^1^ Further research is needed to explore the extent to which the current state of post-publication critique is a result of principled editorial decisions or practical obstacles. It is tempting to look outside of the journal system for solutions to facilitate post-publication critique [7]; however, attempts to establish dedicated platforms have met with limited success—one major platform, PubMed Commons, was shut down in 2018 due to low usage [21]. In box 1, we offer some tentative policy suggestions (based on our opinion) for journals to consider that may facilitate post-publication critique.

Box 1. **Policies journals could consider adopting to facilitate post-publication critique.**

1. Offer at least one option for post-publication critique.
2. Clearly identify and describe options for post-publication critique in instructions to authors.
3. Clearly state whether post-publication critique will be independently peer reviewed. Recognize that the authors of the target article may provide useful feedback, but cannot be considered neutral.
4. Facilitate expedient handling of post-publication critique submissions to ensure timely dissemination of relevant critique to research consumers.
5. Foster a culture of critique. Actively encourage and highlight post-publication critique to the journal's readership, for example, via editorials.
6. Enhance access to and discoverability of post-publication critique: (a) Tag post-publication critique with appropriate meta-data so they can be indexed in third-party databases, websites and referencing software; (b) display prominent links to post-publication critique alongside target articles; (c) make post-publication critique open access.
7. Remove strict length, time-to-submit and reference limits. Judge post-publication critique on a case-by-case basis and promote concise writing via editorial feedback.
8. Ensure transparent reporting of research articles (e.g. sharing of data, analysis code and materials, and adherence to reporting guidelines) to enable informed critique and debate.
9. Adopt a two-tier post-publication critique system. Tier one involves rapid publication of lightly moderated contributions on the journal's website (i.e. web comments). Tier two curates the most informative Tier one contributions and converts them to formal articles (letters) that become a permanent part of the scientific record and provide appropriate academic credit to their authors. For an example, two-tier system, see BMJ Rapid Responses [22].
10. Improve transparency and accountability by hiring an independent editor responsible for handling post-publication critique [23]. Publish all editorial decisions related to post-publication critique, including the number submitted, rejected and published.

### 3.4. Conclusion

The cultivation, documentation and dissemination of post-publication critique is an important part of a healthy and self-correcting research literature. Our study reveals considerable variation in how post-publication critique is handled by journals operating across scientific disciplines. Clinical Medicine had a more active post-publication critique culture than other disciplines; but its journals also imposed the strictest limits. Overall, post-publication critique appears to be tightly controlled and restricted by top-ranked academic journals. At many journals, it was apparently not possible to publish post-publication critique at all, and journals that did offer options for post-publication critique often imposed strict length and time-to-submit restrictions. The post-publication critique we did identify appeared to have negligible impact on target article authors' conclusion. These data provide a stratum of empirical evidence upon which to base debates about how scientific critique should be optimally handled. We encourage stakeholders across the academic ecosystem to explore ways to foster a richer culture of post-publication critique.

## Data Availability

All data, materials and analysis scripts are publicly available on the Open Science Framework (https://osf.io/8b6jy/). To facilitate reproducibility, this manuscript was written by interleaving regular prose and analysis code using knitr [24] and papaja [25], and is available in a Code Ocean container (https://doi.org/10.24433/CO.3805142.v1) which re-creates the software environment in which the original analyses were performed. Electronic supplementary material is available online [26].

## Footnotes

1 Note that these journals could technically claim they are in compliance with this guideline because post-publication critique is always possible on PubPeer, which operates independently of journals. Also note that the International Committee of Medical Journal Editors recommends that ‘Medical journals should provide readers with a mechanism for submitting comments, questions, or criticisms about published articles, usually but not necessarily always through a correspondence section or online forum’.
